# Reversible-gel-assisted, ambient-pressure-dried, multifunctional, flame-retardant biomass aerogels with smart high-strength-elasticity transformation

**DOI:** 10.1093/nsr/nwae360

**Published:** 2024-10-15

**Authors:** Ting Wang, Ying-Jiao Zhan, Ming-Jun Chen, Lei He, Wen-Li An, Shimei Xu, Wei Wang, Jian-Jun Shi, Hai-Bo Zhao, Yu-Zhong Wang

**Affiliations:** College of Chemistry, The Collaborative Innovation Center for Eco-Friendly and Fire-Safety Polymeric Materials (MoE), National Engineering Laboratory of Eco-Friendly Polymeric Materials (Sichuan), State Key Laboratory of Polymer Materials Engineering, Sichuan University, Chengdu 610064, China; Green Preparation and Recycling Laboratory of Functional Polymeric Materials, College of Science, Xihua University, Chengdu 610039, China; Green Preparation and Recycling Laboratory of Functional Polymeric Materials, College of Science, Xihua University, Chengdu 610039, China; Green Preparation and Recycling Laboratory of Functional Polymeric Materials, College of Science, Xihua University, Chengdu 610039, China; College of Chemistry, The Collaborative Innovation Center for Eco-Friendly and Fire-Safety Polymeric Materials (MoE), National Engineering Laboratory of Eco-Friendly Polymeric Materials (Sichuan), State Key Laboratory of Polymer Materials Engineering, Sichuan University, Chengdu 610064, China; College of Chemistry, The Collaborative Innovation Center for Eco-Friendly and Fire-Safety Polymeric Materials (MoE), National Engineering Laboratory of Eco-Friendly Polymeric Materials (Sichuan), State Key Laboratory of Polymer Materials Engineering, Sichuan University, Chengdu 610064, China; College of Chemistry, The Collaborative Innovation Center for Eco-Friendly and Fire-Safety Polymeric Materials (MoE), National Engineering Laboratory of Eco-Friendly Polymeric Materials (Sichuan), State Key Laboratory of Polymer Materials Engineering, Sichuan University, Chengdu 610064, China; Science and Technology on Advanced Functional Composite Laboratory, Aerospace Research Institute of Materials & Processing Technology, Beijing 100076, China; Science and Technology on Advanced Functional Composite Laboratory, Aerospace Research Institute of Materials & Processing Technology, Beijing 100076, China; College of Chemistry, The Collaborative Innovation Center for Eco-Friendly and Fire-Safety Polymeric Materials (MoE), National Engineering Laboratory of Eco-Friendly Polymeric Materials (Sichuan), State Key Laboratory of Polymer Materials Engineering, Sichuan University, Chengdu 610064, China; College of Chemistry, The Collaborative Innovation Center for Eco-Friendly and Fire-Safety Polymeric Materials (MoE), National Engineering Laboratory of Eco-Friendly Polymeric Materials (Sichuan), State Key Laboratory of Polymer Materials Engineering, Sichuan University, Chengdu 610064, China

**Keywords:** biomass aerogel, ambient-pressure drying, flame retardance, solvation-controlled elastification, harsh-condition resistance

## Abstract

Bio-based aerogels, which are poised as compelling thermal insulators, demand intricate synthesis procedures and have limited durability under harsh conditions. The integration of smart stimuli–response transitions in biomass aerogels holds promise as a solution, yet remains a challenge. Here, we introduce a pioneering strategy that employs reversible-gel-assisted ambient-pressure drying without organic solvents to craft multifunctional bio-based aerogels. By exploiting the thermally reversible gelling propensity of select biomasses, we anchor emulsified bubbles within cross-linked hydrogels, circumventing surface tension issues during mild drying. The resultant aerogels feature a robust porous matrix that is imbued with stable bubbles, yielding low thermal conductivity, high flame retardancy and robust resistance to diverse rigors. This innovative approach facilitates a paradigm shift in intelligent fire protection in which aerogels transition from robust to flexible in response to water stimuli, effectively shielding against thermal hazards and external forces. This work opens up a facile, eco-friendly and mild way to fabricate advanced biomass aerogels with stimuli-responsive transformation.

## Teaser

This work demonstrates a reversible-gel-assisted ambient-pressure-drying strategy for crafting multifunctional biomass aerogels with smart high-strength-elasticity transformation for intelligent firefighting thermal insulation.

## INTRODUCTION

The urgency to develop high-performance sustainable insulating materials arises from concern about global warming and the depletion of petrochemical resources [1–4]. Featuring abundant renewable sources, excellent mechanical properties and potential multi-functionalization, biomass aerogels have emerged as promising alternatives for petroleum-based polymer foams [5–9]. These aerogels are constructed by dehydrating a biomass gel or solution [10,11], requiring specific drying processes such as supercritical drying [12] and freeze-drying methods [13,14], which eliminates the negative impacts of surface tension on the microstructures of aerogels during drying [15,16]. Unfortunately, the traditional methods are time-consuming, expensive and challenging to scale up [5,17,18]. In recent years, there has been significant research that has focused on the development of an ambient-pressure drying method [19,20]. Various techniques have been explored to apply hydrophobic treatments and utilize solvent-exchange procedures to mitigate the impact of surface tension on the aerogel microstructure under open conditions [21,22]. Despite these advancements, however, the resultant biomass aerogels require complex procedures that use numerous organic solvents and have poor mechanical strength [23], high flammability [6,24] and weak environmental tolerance [25,26].

The development of high-performance and functional aerogels through a green and facile fabrication approach remains an imperative and challenging task. Zhang *et al.* [27–29] first proposed a hyperbolically structured aerogel with a negative index, offering one of the most popular selections for overcoming the conflict between mechanical and thermal properties. Recently, some researchers proposed to directly cast aerogels by using air bubbles as templates without the need for lyophilization and other modifications [30,31]. By incorporating surfactants or hydrophobic particles at the gas–liquid interface during emulsion, air bubbles can be stabilized in the slurry, enabling the *in situ* drying of suspensions under open conditions to obtain porous aerogels. However, air bubbles are inherently unstable both thermodynamically and kinetically, making them susceptible to damage from drying, liquid drainage and gas diffusion driven by Laplace pressure [32]. The preservation of air bubbles in biomass solutions is crucial for the preparation of advanced biomass aerogels in an open environment. One potential approach to achieving this is by rapidly and significantly transforming biomass solutions that contain air bubbles into gels. Interestingly, certain biomass polymers, such as gelatin, carrageenan and gellan gum, possess the unique ability to form polymer solutions at higher temperatures and transition into hydrogels at lower temperatures, owing to their thermal reversible gel properties. This gelation process occurs as a result of extensive intermolecular and intramolecular forces, leading to the development of 3D gel networks upon cooling [33]. By manipulating the molecular structure and components, it becomes possible to facilitate the green and straightforward fabrication of multifunctional biomass aerogels with exceptional performance.

Herein, we propose a new organic-solvent-free, reversible-gel-assisted, ambient-pressure-dried method for the fabrication of multifunctional biomass aerogels. In this green approach, gelatin with both thermo-reversible gelling capacity and air-bubble-stabilizing ability was used to build the scaffold, while the melamine formaldehyde resin was incorporated as a cross-linking agent and functional component. The fabrication process involved intense stirring of the polymer solution to generate numerous gelatin-stabilized air bubbles at elevated temperatures, which can be stably immobilized within the hydrogel through rapid cooling for subsequent drying under ambient pressure and mild conditions. This method enables the ambient-pressure-dried production of biomass aerogels with remarkable characteristics, including a high modulus (≤5.5 MPa), low thermal conductivity (30.8 mW m^−1^ K^−1^), excellent resistance to harsh conditions including various solvents and a wide temperature range from –20 to 100°C, flame retardancy with a high limiting oxygen index (LOI) of 36.5% and smart stimulus–response firefighting. Remarkably, benefitting from steady chemical and reversible physical cross-linking structures, the aerogel manifested unique different solvation-controlled responses. When exposed to water, the robust aerogel can reversibly transform into elastic wet gels by absorbing water. This elastic wet gel can effectively resist the continuous external impact of a high-temperature flame (1400°C) and maintain a low back temperature of 80°C, providing sufficient escape conditions for fire rescue. This feature opens up a new strategy for thermal insulation materials in intelligent firefighting.

## RESULTS AND DISCUSSION

### Material synthesis and morphology

**Scheme 1.**
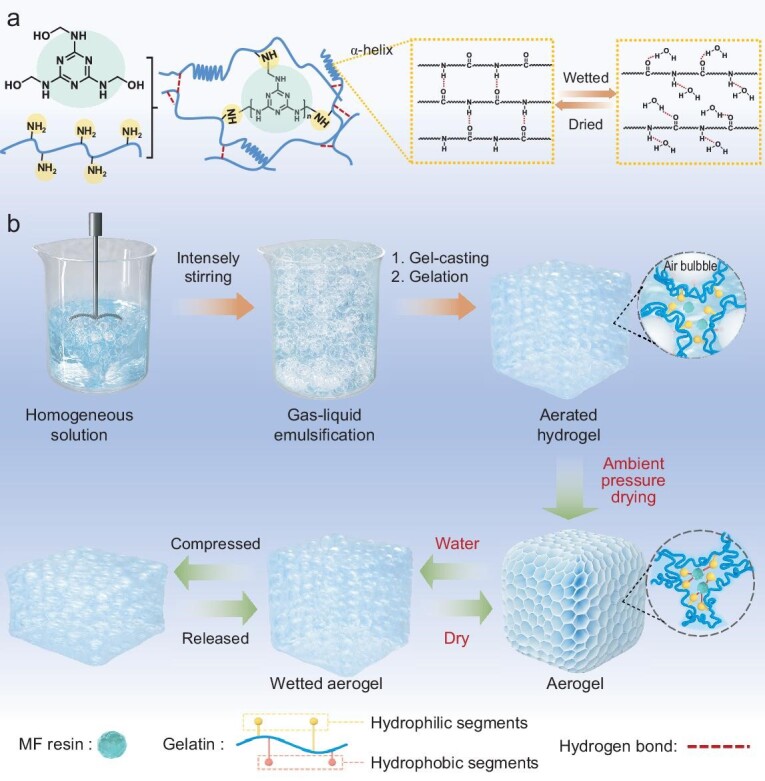
Material synthesis. (a) Chemistry behind the fabrication and (b) illustration of the synthesis process of G10Mn aerogels with an inter-cross-linked infrastructure.

**Figure 1.**
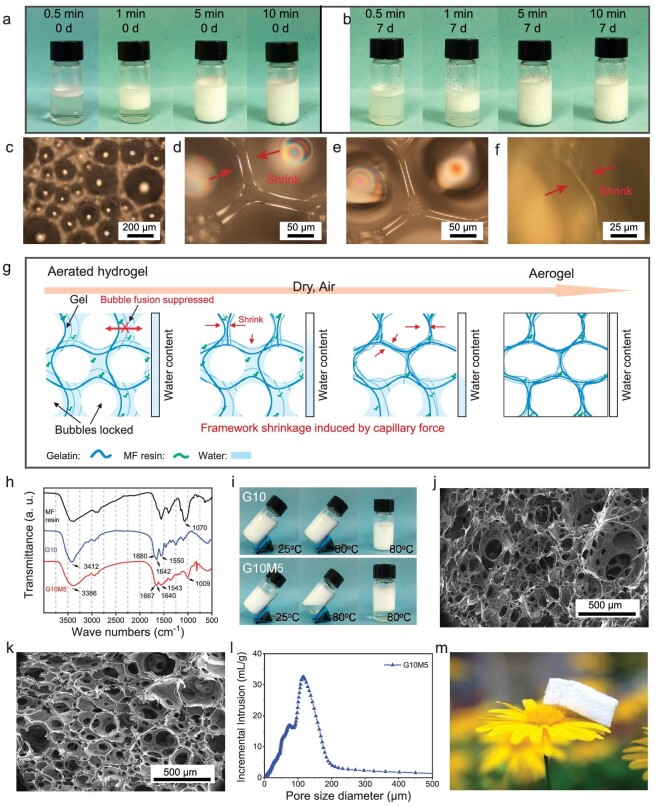
Drying processes and structures of aerogels. Images of G10M5 aqueous foam prepared by using different emulsification times after (a) 0 d and (b) 7 d. (c) and (d) POM images of G10M5 aerated hydrogel without drying. POM images of G10M5 aerated hydrogel after being dried at room temperature for (e) 30 min and (f) 4 h. (g) Schematic illustration of structural evolution during ambient-pressure drying processes. (h) FTIR spectra. (i) Images of G10 and G10M5 aerated hydrogels before and after heating at 80°C. SEM images of (j) G10 and (k) G10M5. (l) Pore size diameter distribution of G10M5. (m) Image of G10M5 standing on the petals.

The synthetic processes of the aerogels are presented in Scheme 1. The reversible-gel-assisted surfactant-biomass foaming method for fabricating aerogels under ambient conditions is developed based on the following criteria. (i) Air bubbles are used as templates to construct aerogels with 3D porous structures, avoiding structural collapse and catastrophic shrinkage at the liquid–solid interface. (ii) The biomass scaffolds should have both a thermo-reversible gelling capacity and foam-stabilizing ability. In this way, the air bubbles can be stabilized in the mixed solution at a higher temperature by intensive stirring. Then, the obtained mixture can instantly turn into a gel (called aerated hydrogel) once exposed to a low temperature, for preserving air bubbles as much as possible. (iii) The flame retardant is used as the cross-linking agent to build a stable cross-linking network and endow the aerogel with high mechanical strength as well as flame retardance. (iv) To endow the aerogel with responsive elasticity, it requires a suitable balance between chemical and physical cross-linking structures. The thermo-reversible biomass undergoes a disorder-to-order chain arrangement transition, allowing the aerogels to adjust their chain mobility through hydrogen bonds. Additionally, the presence of chemical cross-linking points prevents the chains from slipping after compression [34]. To meet these requirements, gelatin with both foam-stabilizing ability and thermo-reversible gelling capacity was chosen as the scaffold for the GM aerogel and water-soluble melamine formaldehyde (MF) resin with reactive functional groups was used as the cross-linking and flame-retardant agent (Scheme 1a). The gelatin/MF solution can be foamed at a comparatively high temperature (>40°C) and rapidly gelled at room temperature to preserve the air bubbles promptly (Fig. S1). The foaming volume is proportional to the stirring time (Fig. 1a). After 10 min of foaming, the volume of the solution expanded by almost five times. As shown in Fig. 1b, the air bubbles in the aerated hydrogel can be well preserved for >7 d. The aerated G10M5 hydrogel with a lower density can even float in water (Fig. S2). With the drying process under normal pressure, the chemical cross-linking between the MF and the gelatin occurred further, strengthening the gel network skeleton. Thus, the air bubble can be kept in the scaffold to achieve the construction of the aerogel under mild conditions.

To demonstrate the advantages of the reversible-gel-assisted and ambient-pressure-dried method, the normal hydrogel and aerated hydrogel were used to prepare the corresponding xerogel and aerogel, respectively (Fig. S3). Due to the high capillary pressure, the xerogel exhibited significant volume shrinkage and the porous structures disappeared after drying at ambient pressure. On the contrary, benefitting from air bubble templates, the reversible gel-assisted foaming method enables the aerogel to retain pores significantly even after direct drying under an open condition. The morphology evolution during the drying process was observed *in situ* by using polarized-light optical microscopy (Fig. S4 and Fig. 1c–f) and the corresponding conclusion is illustrated in Fig. 1g. The even dispersion of air microbubbles in the aerated hydrogel (Fig. 1c) is the key factor for obtaining an aerogel with continuous pore structure. In a common situation, gas bubbles tend to diffuse from small bubbles to large adjacent bubbles due to the influence of Laplace pressure. In this work, the gelatin gel-locked bubbles can be preserved throughout the entire drying process. When adding MF, under the cross-linking action between the MF and the gelatin, the diameters of the air bubbles were changed from 100–300 μm (G10) to 300–500 μm (G10M5). Notably, after being dried for 30 min, the thickness of the bubble walls became thinner during the removal of the water from the aerated G10M5 hydrogel (Fig. 1d–f). Within the gelatin framework, driven by capillary tension and strong hydrogen bonding between molecular chains, the framework continuously becomes thinner during water evaporation.

The chemical and physical cross-linking of the aerogel was demonstrated by using FTIR spectra (Fig. 1h). In the amide I band (Fig. S5), G10M5 manifested reduced β-sheet (1633 cm^−1^) and β-turn absorption, whereas strong α-helix (1650 and 1658 cm^−1^) and random coil (1643 cm^−1^) structure absorption could be observed compared with G10 [34,35]. The stretching and bending vibrations of N–H in G10M5 moved to lower wave numbers of 3386 and 1543 cm^−1^ from 3412 and 1550 cm^−1^ (neat gelatin), indicating that abundant H-bonds were formed after the MF resin was added [36]. The bending vibration of the –OH of the hydroxymethylated melamine (1070 cm^−1^) disappeared, indicating that chemical cross-linking processes occurred between the MF resin and the gelatin [5,17,37]. The gelation phenomenon of the G10M5-aerated hydrogel was observed after being heated to 80°C, while the neat gelatin-aerated hydrogel with thermo-reversible gelling capacity turned into a liquid (Fig. 1i). From the above, G10M5 with chemical and physical cross-linking networks was formed. Scanning electron microscopy (SEM) was used to observe the microstructures of the aerogels (Fig. 1j and k). Both G10 and G10M5 manifested typical bubble-filled porous structures. For G10, a large number of polar groups, such as amino, carboxyl and hydroxyl groups, would form strong physical interactions during the drying process, resulting in large shrinkage, small pore size and wide pore size distribution. In contrast, the MF resin reduced the number of polar groups as well as the foaming capacity of the gelatin, thus forming larger air bubbles (Fig. 1c). The significantly increasing viscosity of the MF/gelatin solution prevented the diffusion of inter-bubble gas before gelation. Consequently, the G10M5 aerogel exhibited a narrow pore distribution and a larger pore size. From the results of mercury intrusion porosimetry (Fig. 1l), the pore size of G10M5 was in the range of 50–200 μm and the average pore diameter was 36 μm. The porosity of G10M5 was 73.60%. In addition, with a low density of 0.077 g/cm^3^, G5M10 can stand on the petal (Fig. 1m).

### Mechanical performance, thermal insulation and harsh-condition resistance

**Figure 2.**
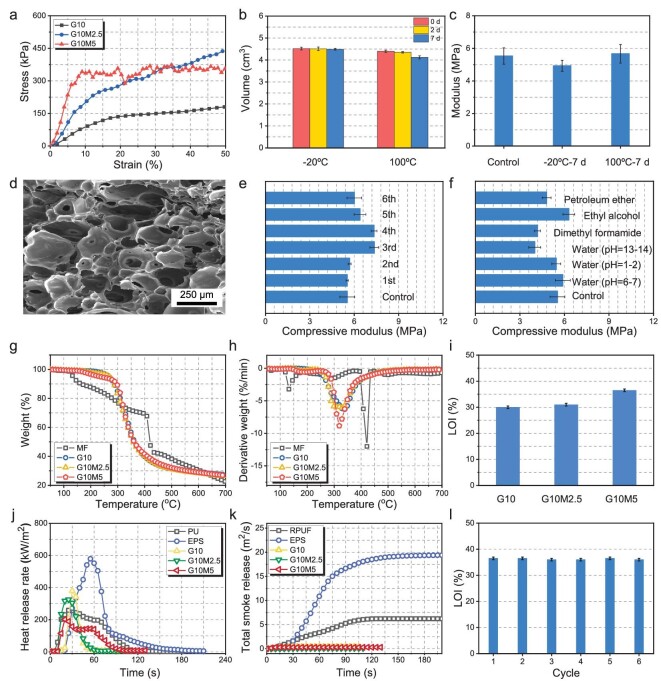
Mechanical properties and flame retardance. (a) Compressive stress‒strain curves of aerogels. (b) Dimensional and (c) mechanical stability of G10M5. (d) SEM image of G10M5 after being soaked in water at 90°C six times. (e) Compressive modulus of G10M5 after being soaked in water at 90°C and dried at 60°C six times. (f) Compressive modulus of G10M5 after being soaked in different solvents at room temperature for 7 d. (g) TG and (h) Derivative thermogravimetry curves of samples. (i) LOI values of aerogels. (j) pHRR and (k) total smoke release curves. (l) LOI values of G10M5 after being soaked in water at 90°C and dried at 60°C six times.

The chemical/physical double cross-linking networks as well as homogeneous pore structures significantly enhance the mechanical properties of the aerogel. As shown in Fig. 2a and Table S2, the compressive strength and elastic modulus increased with increasing amounts of MF resin. The elastic modulus of G10M5 was high at 5.53 MPa, which was almost four times that of G10 (1.4 MPa). G10M5 manifested a compression strength of 335 kPa, which is higher than that of G10 (87 kPa) and traditional petroleum-based foams, such as expanded polystyrene foam [38] (EPSF, 70 kPa) and rigid polyurethane foam (RPUF, 200 kPa) [39]. The thermal conductivities of aerogels at room temperature are listed in Table S3. It was obvious that, with the increasing addition of the MF resin, the thermal conductivities of the aerogels decreased from 42.0 mW m^−1^ K^−1^ (G10 aerogel) to 30.8 mW m^−1^ K^−1^ (G10M5 aerogel). Combined with the SEM images (Fig. 1j and k), the large pores of the G10 aerogel were heat-penetrative, while the narrower-pore-diameter distribution of G10M5 can decrease heat dissipation. The thermal conductivity of G10M5 is comparable to that of traditional petroleum-based foams, such as EPS (35–45 mW m^−1^ K^−1^) and RPUF (30 mW m^−1^ K^−1^). With higher mechanical stiffness, comparable thermal insulating properties and an eco-friendly manufacturing method, the G10M5 aerogel manifests superior advantages compared with traditional petroleum-based foams.

Figure 2b and c illustrates the exceptional dimensional and mechanical stability of the G10M5 aerogel under different harsh conditions. Even when subjected to high/low temperatures for a long time, the aerogel maintained its original volume at –20°C, with only slight volume shrinkage observed at 100°C (Fig. 2b). The G10M5 aerogel displayed remarkable mechanical stability in harsh environments, as evidenced by its nearly unchanged modulus (≥5 MPa) after exposure to –20°C and 100°C for 7 days (Fig. 2c). In general, the water sensitivity largely restricts practical applications of aerogels. To study the water resistance of the aerogels, G10M5 was subjected to repeated soaking in hot water at 90°C for 6 h and re-drying at 60°C in an open system, and the corresponding data are listed in Fig. 2d and e, Fig. S6 and Table S4. After being soaked in water at 90°C, G10 was dissolved, while G10M5 still retained its original shape with slight swelling. It was worth noting that, after immersion and drying were repeated six times, the sample presented a complete cellular structure (Fig. 2d). During the cyclic treatment, the aerogel showed a similar modulus (>5 MPa) to the original one (Fig. 2e). That is, the special bubble-filled porous structures of G10M5 could resist the negative effects that were made by the high surface tension of the water during the repetitive drying process. Notably, even after being treated with solvents of different polarity and pH values (Fig. 2f and Table S5), G10M5 still showed excellent property stability. Thermogravimetric analysis (TGA) was conducted to characterize the thermal stability of the aerogels (Fig. 2g and h, and Table S6). The MF resin exhibited a small weight loss peak (100–200°C) due to the dehydration of the hydroxyls. In contrast, no peak appeared in this temperature range for G10M2.5 and G10M5, further demonstrating that a chemical cross-linking reaction existed between the amino groups of the gelatin and the hydroxyls of the MF resin. The introduction of MF resin slightly reduced the initial decomposition temperature and maximum decomposition temperature from 267.6°C and 336.0°C (G10) to 235.2°C and 318.8°C (G10M5), respectively. In addition, the weight loss rate increased with increasing MF resin content, due to the high mass loss rate of the MF resin with the release of NH_3_ according to our previous work [5,17]. With good stiffness, excellent mechanical stability and thermal stability, the GM aerogel that is prepared by using a facile manufacturing method is highly favorable for practical applications.

The high flammability of conventional porous thermal insulators poses significant risks to both properties and human safety. The flame retardancy of G10M5 was evaluated by using LOI, a vertical burning test (UL-94) and a cone calorimetry test. As shown in Fig. 2i and Table S7, the LOI values of the aerogels increased with the introduction of the MF component. G10M5 exhibited an LOI value as high as 36.5% and achieved the highest vertical burning rating (V-0), whereas the neat gelatin aerogel (G10) failed to pass the vertical burning tests. The cone calorimetry results (Fig. 2j and k, Fig. S7 and Table S8) revealed a gradual reduction in the peak heat release rate (pHRR) as the amount of MF resin increased. G10M5 manifested a pHRR of 216.3 kW/m^2^, which is a 46.8% drop compared with G10. It is lower than the traditional insulating foams, such as RPUF (258.6 kW/m^2^) and EPS (602.2 kW/m^2^), indicating better fire safety. The aerogel also exhibited great smoke suppression behaviors (Fig. 2k). All samples manifested a total smoke release of 0.1–0.39 m^2^, which is far lower than that of RPUF (7 m^2^) and EPSF (20 m^2^). When exposed to a torch flame with a maximum temperature of 1400°C for 5 min, the G10M5 material did not ignite. In addition, the durability of the flame retardancy of the aerogel was assessed by subjecting the samples to repeated soaking–drying cycles in water at 90°C. Figure 2l demonstrates that G10M5 maintained an LOI value of 36%, indicating excellent flame-retardant stability. This remarkable performance can be attributed to the steady bonding between the flame-retardant (MF resin) and the amino groups of the gelatin. The outstanding flame retardance of G10M5 primarily arises from the gas phase flame retardance of the MF resin, which can release a substantial amount of noncombustible gas (NH_3_) [5,40].

### Reversible solvation-controlled elastification

**Figure 3.**
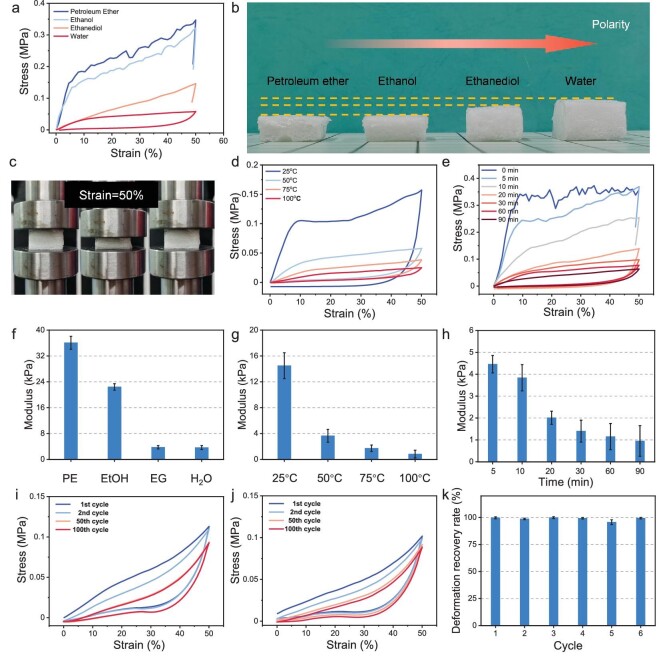
Reversible solvation-controlled elastification. (a) Stress‒strain curves of G10M5 after being soaked in solvents at room temperature for 7 d. (b) Digital images of aerogels soaked in solvents after compression tests. (c) Digital images of compression test of wetted aerogels. Stress‒strain curves of G10M5 after being soaked in water for different (d) temperatures and (e) times. Compression modulus of G10M5 after soaking in (f) solvents at room temperature for 7 d, (g) water at different temperatures for 30 min and (h) water at 80°C for different times. Compression and release processes of G10M5 after being soaked at 90°C (i) once, (j) six times and (k) its deformation recovery ratio.

The aerogel possesses distinct and reversible responses to various solvents, enabling precise control over their physical properties. In our design, the thermo-reversible gelling property of gelatin is facilitated by the transformation of its random coil structure into helices through hydrogen bonding. Thus, the reversible physical interactions lead to the stimulus–response transitions of the G10M5 aerogel. Various solvents, such as petroleum ether, ethanol, ethanediol and water, were employed to disrupt the strong interchain physical bonds to impart mobility to the polymer chains of the aerogel. The mechanical properties of wetted G10M5 aerogels were evaluated after immersion in solvents with different polarities, as depicted in Fig. 3a. Upon soaking in petroleum ether and ethyl alcohol (ETOH), a sharp decrease in compressive strength was observed for the wetted G10M5 aerogels (Fig. 3a). However, as the polarity of the soaking solvents increased, the rigid nature of G10M5 transformed into elasticity. The shape recovery of the G10M5 aerogels improved with the increasing polarity of the solvents that were used for soaking (Fig. 3b). When soaked in water, the wetted G10M5 aerogel exhibited high elasticity and was capable of fully recovering its original shape after the stress was removed (Fig. 3c). The elasticity of the wetted G10M5 aerogel was evaluated by soaking it in water at different temperatures and durations (Fig. 3d and e). The strain–stress curves exhibited reduced shape deformations and hysteresis ring areas with increasing soaking temperatures. After being soaked in water at 80°C for 5 min, the toughness of G10M5 increased (Fig. 3e). Continued soaking for >20 min transformed the wetted G10M5 from a rigid state, displaying excellent resilience. With increasing soaking times and temperatures, the mechanical strength decreased gradually (Fig. 3f–h). Specifically, the modulus of G10M5 soaked in ethylene glycol (EG) and water sharply decreased to 3.74 and 3.65 kPa, respectively, from its initial value of 5.5 MPa in the dry state. This decrease can be attributed to the polar solvents, such as water and EG, which significantly broke the hydrogen bonds between the polymer chains. Increases in the soaking temperature and duration accelerated the disruption of the hydrogen bonds, resulting in reduced rigidity and increased elasticity of the wet aerogels.

To assess the stability of the solvent-induced transformations, repeated wetting-and-drying cycles were performed on the G10M5 aerogels. The G10M5 aerogel was soaked in hot water at 90°C and subsequently dried under open conditions at 80°C. As shown in Fig. 3i–k, after a single wetting-and-drying cycle, the wetted aerogel exhibited the capability to withstand compression of ≤50% deformation without cracking and could fully recover its original shape. Even after undergoing six repeated wetting-and-drying cycles, the aerogel maintained its resilient performance, with stress–strain curves that were similar to that in the initial cycle. The deformation recovery ratio is recorded in Fig. 3k, demonstrating that, even after being subjected to cyclic wetting and drying, the wetted G10M5 aerogel retained a deformation of as low as 2%. The remarkable durability of the simulated transformation provides opportunities for achieving long-term smart functionality.

**Figure 4.**
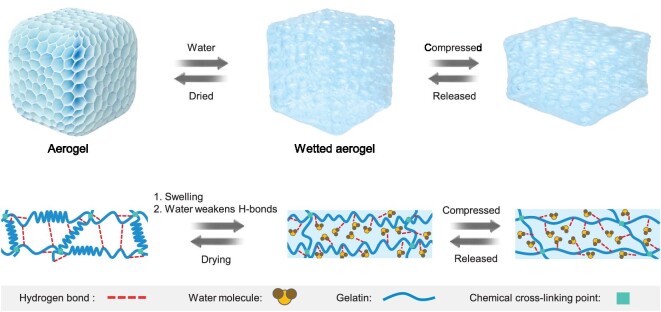
Elasticity transfer mechanism. Schematic diagram of the elasticity transfer of G10M5 aerogel.

The solvation-controlled elastification mechanism is depicted in Fig. 4. Initially, water at a high temperature exerts a significant weakening effect on the strong interchain hydrogen bonds between the polymer chains. The aerogel swells when exposed to such high-temperature water, resulting in the filling of spaces between the polymer chains with abundant water molecules. This process promotes the rapid conversion of α-helix structures into random coils. Consequently, a substantial number of strong polymer–polymer hydrogen bonds are broken, while weak polymer–water hydrogen bonds are formed [41,42]. Additionally, the unique bubble-like pore structures within the aerogel provide ample space for the frameworks of the wetted aerogel to deform. These factors collectively enable the polymer chains to exhibit increased mobility during compression, resulting in a decrease in the compression strength of the wetted aerogel. Moreover, the networks of G10M5 are effectively cross-linked by the rigid MF resin, thereby reducing the overall deformation of the network. As a result, the wetted aerogel can efficiently recover its original shape upon the removal of stress. More importantly, when the wetted aerogel is cyclically dried, strong polymer–polymer hydrogen bonds can be reformed and re-broken accordingly, while the chemically cross-linked network is preserved throughout the process. Therefore, the aerogel manifested a unique reversible resilient performance during the repetitive wetting-and-drying processes.

### Smart stimulus–response firefighting

**Figure 5.**
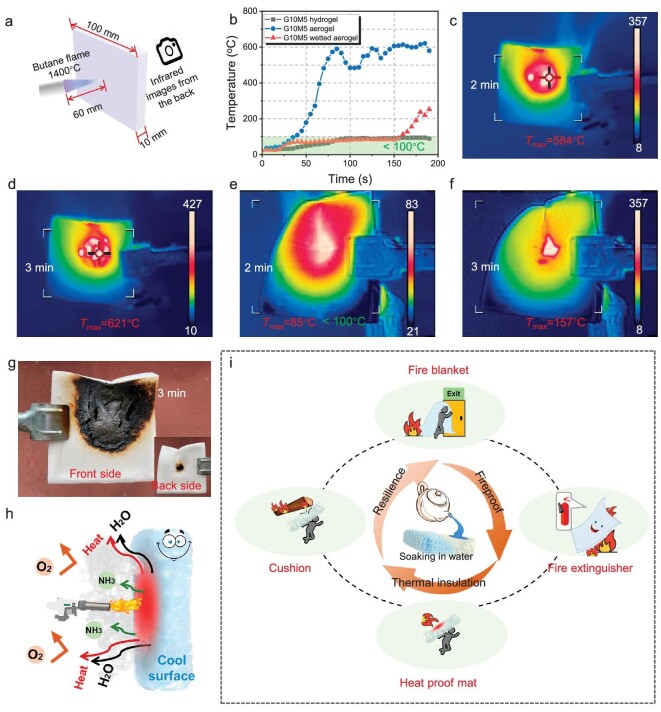
Thermal insulation and smart stimulus–response firefighting. (a) Schematic diagram of the fire-resistance test of the aerogel. (b) Curves of maximum temperature on the backside of the samples treated with butane flame at 1400°C for different times. (c and d) Infrared images of the back side of the G10M5 aerogel burnt at different times. (e and f) Infrared images of the back side of the wetted G10M5 aerogel burnt at different times. (g) Digital images of the wetted G10M5 aerogel after the burning test. (h) Fire-resistant mechanism of the wetted G10M5 aerogel. (i) Versatile applications of GM aerogel.

The stimuli–response transformation from strong aerogels to elastic hydrogels provides a new idea for intelligent fire protection. Hydrogels that are composed of a large amount of water (high specific heat capacity and enthalpy of vaporization) can be used to extinguish fire by cooling the temperature of combustibles and releasing a large amount of non-flammable water vapor, while high elasticity can prevent physical impact damage. By the same token, the wet aerogel with a flame-retardant polymer scaffold and smart stimulus–response resilience can be used as a fire extinguisher and protector. To prove it, the high-temperature thermal insulating performance of the wetted aerogels was evaluated by using a burning test (Fig. 5a). In brief, the wetted G10M5 aerogel with dimensions of 10 mm × 100 mm × 100 mm was obtained by immersing the aerogel in water at 80°C for 2 h, which was continuously burnt by using a butane flame with a temperature of 1400°C. The backside temperature changes of the G10M5 aerogel, G10M5 hydrogel and wetted G10M5 aerogel were recorded by using an infrared imaging device (Fig. 5b–f and Fig. S8). During the first 50 s of the burning test, the temperature of the wetted G10M5 gradually increased from 27°C to 75°C, which was much lower than the temperature of the G10M5 aerogel (180°C) and comparable to that of the G10M5 hydrogel (55°C). What is more, the backside temperature of the wetted G10M5 remained at <100°C for ≥150 seconds (Fig. 5b and d_1_), while the G10M5 aerogel was burnt through, and the backside temperature reached >500°C (Fig. 5c_1_). That is, almost 95% of the heat transfer was blocked by the wetted aerogel, which is highly superior to every other report. Excellent high-temperature thermal insulation ability can protect people from thermal hazards in a fire. Finally, the temperature sharply increased after burning for 150 s due to the penetration of the sample by the flame (Fig. 5c and d). Even so, the heat was mainly concentrated in a small area where the wetted aerogel was in direct contact with the flame, while other parts remained cold throughout the test. With higher water content, the backside temperature of the G10M5 hydrogel can remained at ∼90°C throughout the whole test (Fig. S8).

The excellent high-temperature fire resistance is ascribed to the flame-retardant polymer scaffold and adequate water content (Fig. 5f). In the early stage of the burning test, the water in the wetted aerogel evaporated and absorbed a large amount of heat, due to the high specific heat capacity and vaporization enthalpy [43–45]. In addition, the formed water vapor can dilute the oxygen concentration on the surface of the sample [44]. As a result, the temperature of the material drops and the fire is extinguished. When the water of the burning part runs out after a prolonged time, the dried flame-retardant scaffold turns into firmly integrated char, which can further stop the spread of the fire. Therefore, the sample remained un-ignited throughout the entire burning test. The continuous pore structure can further enhance the thermal insulation of the wetted aerogel [43,45].

The versatile applications of the GM aerogel in intelligent fire protection are illustrated in Fig. 5g. With its exceptional mechanical strength and flame-retardant properties, this aerogel serves as an ideal thermal insulation material for various settings, including buildings. In the event of a fire, the resilient aerogel transforms into a flexible wet gel upon sustained exposure to water. This dual functionality not only shields individuals from thermal risks, but also buffers against external pressures. If the water source can be continuously replenished, then the aerogel will always insulate the heat and keep the back temperature extremely low. Consequently, the remarkable responsiveness of the GM aerogel in firefighting scenarios underscores its promising and diverse range of potential applications.

## CONCLUSION

In summary, a reversible-gel-assisted and ambient-pressure-dried method was demonstrated to prepare biomass aerogels under mild and green conditions. Through this method, gelatin with both thermo-reversible gelling capacity and air-bubble-stabilizing ability was used to build the scaffold, while the MF resin was incorporated as a cross-linking agent and functional component. The distinctive thermo-reversible gelling capability of gelatin plays a crucial role in entrapping air bubbles within the matrix, setting the stage for the subsequent ambient-pressure drying process. The resulting biomass aerogel with a continuous bubble-filled porous structure exhibited a compressive modulus of 5.53 MPa, low thermal conductivity of 30.8 mW m^−1^ K^−1^ and excellent resistance to various harsh conditions (different polar solvents, strong acid/base solutions and −20°C to 70°C for 7 d). Moreover, the aerogel exhibits impressive flame-retardant properties, as evidenced by a high LOI of 36.5%, self-extinguishing capacity, low heat release (216.3 kW/m^2^) and a negligible smoke production rate (0.056 m^2^/s). Remarkably, owing to its reversible physical and robust chemical cross-linking networks, coupled with its uninterrupted pore structures, the aerogel displayed remarkable resilience from high strength throughout repeated cycles of wetting and drying. The transition from robust aerogels to flexible hydrogels in response to water stimuli introduces a novel concept for intelligent fire protection. The resilient wetted aerogel demonstrates remarkable resistance to high-temperature conditions, maintaining a rear surface temperature of just 80°C even when subjected to a butane flame for 110 s. By capitalizing on its distinctive solvation-controlled reactions and flame-retardant properties, the durable aerogel finds utility as a thermal insulator in typical scenarios. In the event of a fire, it can seamlessly shift into an elastic wet fire blanket through immersion in water, thereby safeguarding individuals from thermal risks and mitigating external force impacts. This study introduces a straightforward and environmentally friendly approach to crafting advanced biomass aerogels that are endowed with multifaceted functionalities.

## METHODS

### Materials

Gelatin that was derived from pork skin was obtained from Sigma-Aldrich Chemical Reagent Corp. Melamine and formaldehyde (37 wt% aqueous solution) were supplied by Kelong Chemical Reagent Corp. (Chengdu, China). All reagents were of analytical grade and used without further purification.

### Synthesis of cross-linked gelatin aerogels

A 10-wt% gelatin solution was prepared by dissolving 10 g of gelatin into 100 mL of double-distilled water at a temperature of 80°C for 30 min. The MF resin precursor solution with a solid content of 50 wt% was prepared according to our previous work [3,13]. Specifically, 5 g of melamine and 10 g of formaldehyde solution were mixed and stirred intensely at 80°C until the melamine was completely dissolved. The detailed formulation is listed in Table S1. The MF resin precursor solution was mixed with 10-wt% gelatin solution at different mass ratios. Typically, 100 mL of a mixed solution at 40°C was stirred at 12 000 r/min for 10 min to obtain the emulsified foam with a foaming ratio of ∼5:1. The wet foam was then placed in the mold and sealed for 24 h at room temperature to obtain the foam gel with chemical cross-linking networks. The gel was dried at 25°C, 60°C and 80°C for 24, 6 and 6 h in an oven with atmospheric pressure, respectively. Finally, the aerogel with stable properties was completed by curing at 80°C for 24 h in a vacuum oven. The obtained aerogel is named the GmMn aerogel, where G and M represent gelatin and MF, and m and n correspond to the corresponding mass ratios, respectively.

### Characterizations

The density of the aerogel was calculated by using the volume and weight of the aerogels. Fourier transform infrared spectroscopy was recorded by using a Nicolet FTIR 170SX spectrometer. The morphological microstructure of the sample was characterized by using SEM (Phe-nom ProX). The images of emulsified foam before and after drying were observed by using a Nikon ECLIPSE LV100 POL polarizing optical microscope (POM). The pore distribution was recorded by using AutoPore Iv 9510 Mercury Intrusion Porosimetry (MIP, Micromeritics Instrument Co., USA). The compression and resilience properties were characterized by using a universal testing machine (Model 3345B13833, Instron Engineering Corporation, USA) at a strain rate of 50 mm min^−1^. The wet aerogels were obtained by soaking in solvents and excess adsorbed solvents in pores were removed. The thermal stabilities of the aerogels were measured by using a TG 209F1 (NETZSCH, Germany) at a heating rate of 10°C min^−1^ in a N_2_ flow of 20 mL min^−1^. According to ASTM D 2863–2009, the LOI values were measured by using an HC-2C oxygen index meter (Jiangning, China). The combustion behaviors were characterized by using a cone calorimeter device (Fire Testing Technology, UK). The specimens were exposed to a heat flux of 50 kW/m^2^ with a size of 100 mm × 100 mm × 10 mm. The thermal conductivity was characterized by using a Hot Disk instrument (TPS2500S) with the transient-plane source method. The infrared images of the aerogels were recorded by using an FTLR T420 infrared thermal camera.

## Supplementary Material

nwae360_Supplemental_File
