# Supplementary material for: Reversible-gel-assisted, ambient-pressure-dried, multifunctional, flame-retardant biomass aerogels with smart high-strength-elasticity transformation
Source: Natl Sci Rev. 2024 Oct 15;11(11):nwae360. doi: 10.1093/nsr/nwae360 (PMC11558063; doi:10.1093/nsr/nwae360)
Supplement: nwae360_Supplemental_File [file nwae360_supplemental_file.pdf]

Supplementary Materials for

Reversible-gel-assisted, ambient-pressure-dried, multifunctional, flame-retardant biomass aerogels with smart high-strength-elasticity transformation

Ting Wang [1, 2], Ying-Jiao Zhan [2], Ming-Jun Chen [2], Lei He [1], Wen-Li An [1], Shimei Xu [1], Wei Wang [3], Jian-Jun Shi [3], Hai-Bo Zhao [1, *], Yu-Zhong Wang[1, *]

*Corresponding author. Email: haibor7@163.com, polymers@vip.126.com

This PDF file includes:

Figs. S1 to S8
Tables S1 to S8

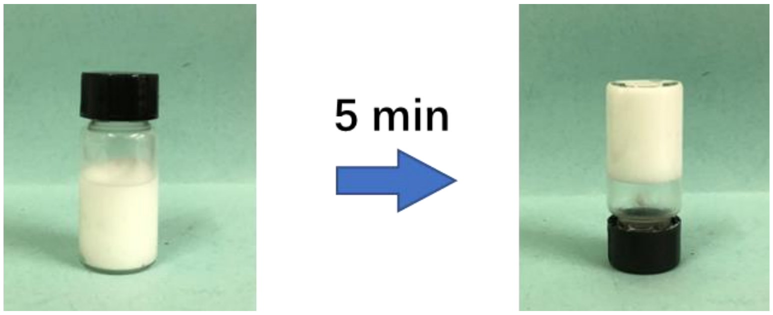

Fig. S1. G10M5 aqueous foam became a gel only after 5 min

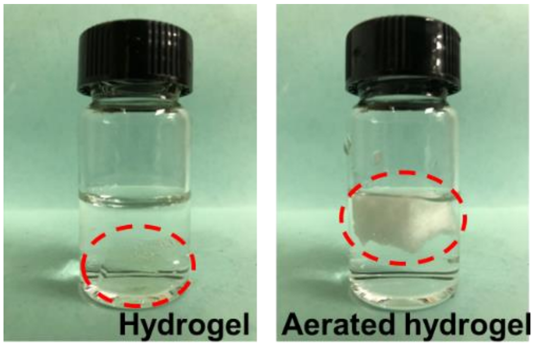

Fig. **S2.** Digital images of G10M5 hydrogel and aerated hydrogel.

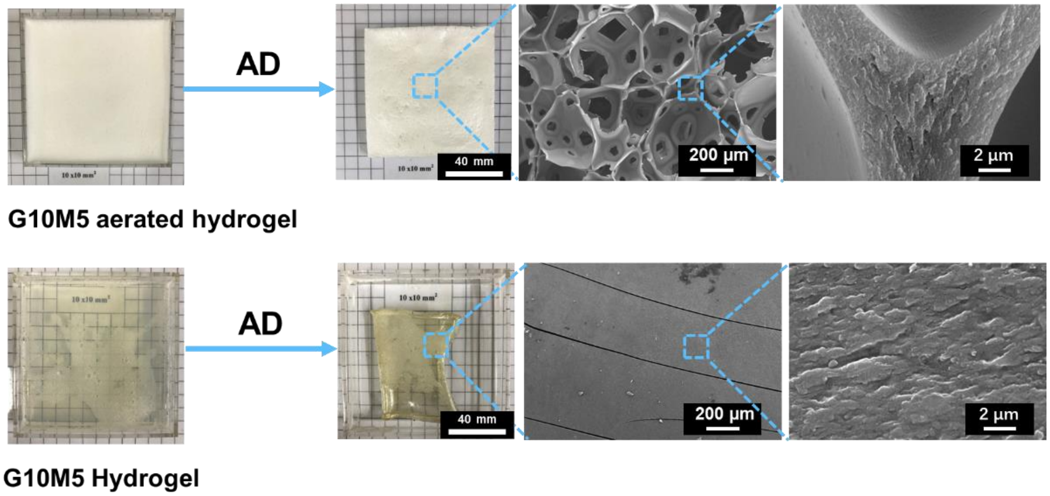

Fig. S3. Digital images and SEM images of samples obtained from different drying processes of hydrogels and aerated hydrogel.

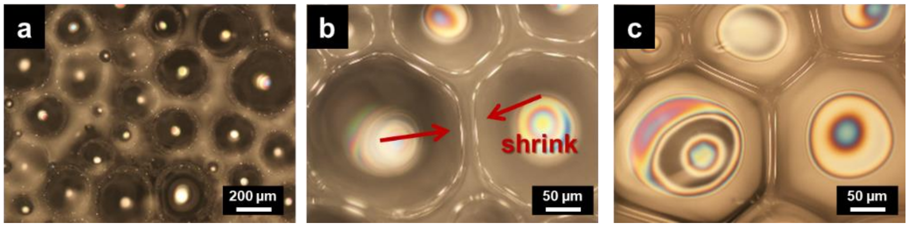

Fig. S4. (a and b) POM images of G10 aerated hydrogel without drying. (c) POM images of G10 aerated hydrogel after being dried at room temperature for 30 min.

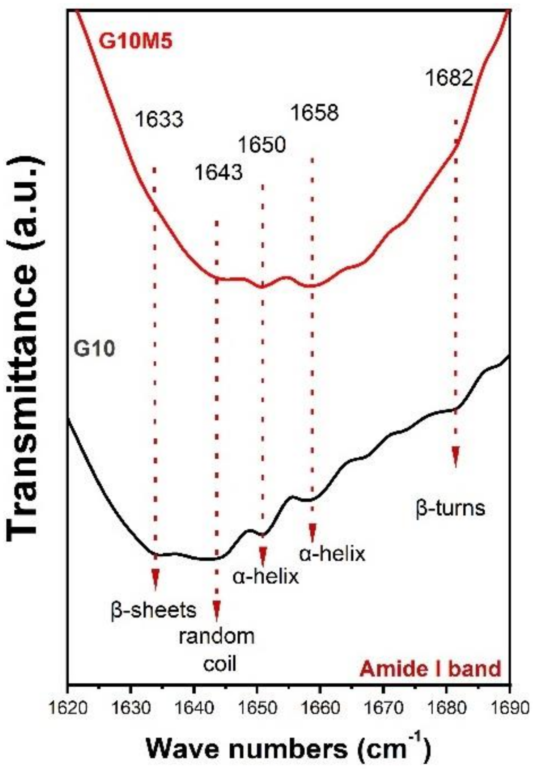

Fig. **S5.** FTIR spectra of G10 and G10M5 in the amide I region.

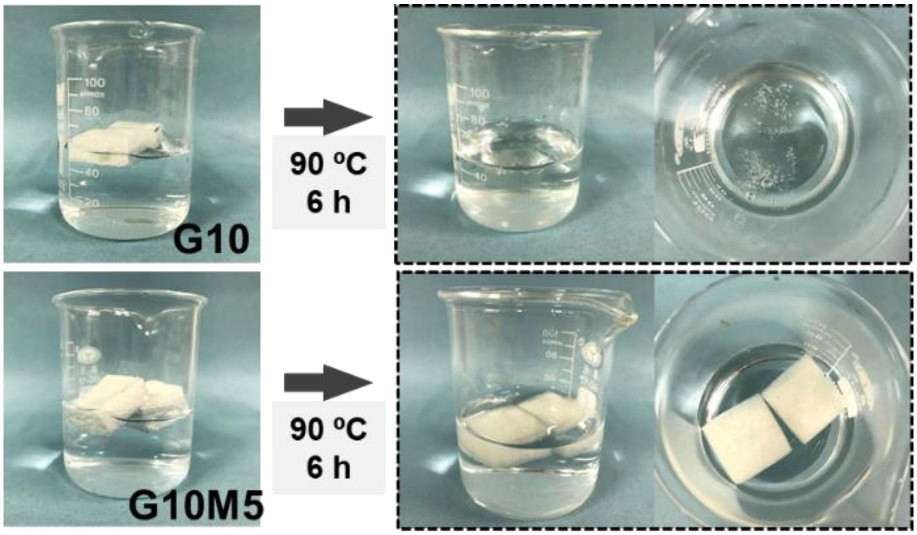

Fig. **S6.** Digital images of G10 and G10M5 soaked in water at 90 ℃.

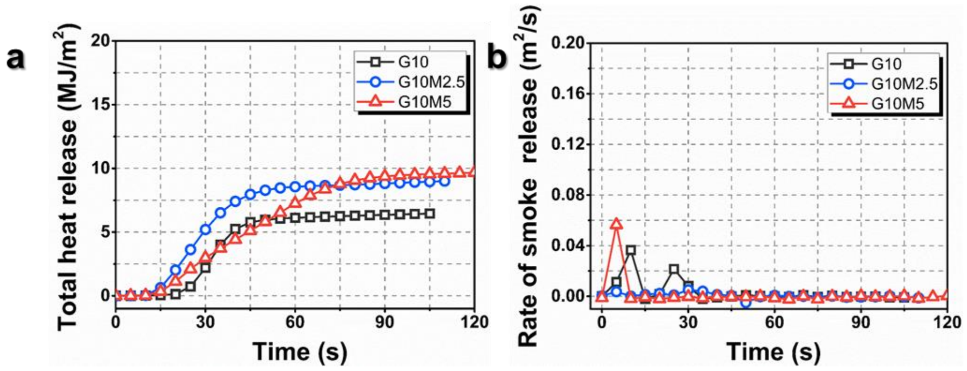

Fig. **S7.** (a) THR and (b)TSR curves of aerogels under a heat flux of 50 kW/m^2

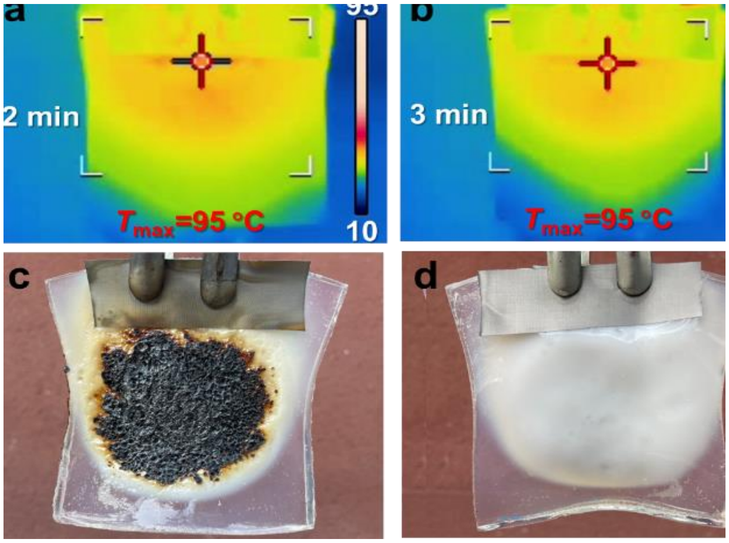

Fig. **S8.** (a and b) Infrared images of the back side of the G10M5 hydrogel at different times. (c and d) Digital images of wetted G10M5 aerogel after the test.

Table S1. Fabrication formulation of aerogels.

Samples	Water (mL)	Gelatin (g)	Melamine (g)	37 wt % Formaldehyde solution (mL)
G10	90	10.000	-	-
G10M2.5	90	10.000	1.667	3.325
G10M5	90	10.000	3.335	6.65

Table S2. Density and mechanical properties of aerogels and organic thermal insulators

Samples	Density (mg cm^{-3})	Modulus (MPa)	Compressive strength (kPa)
G10	40.8 ± 0.2	1.4 ± 0.1	87 ± 20
G10M2.5	51.1 ± 0.2	3.0 ± 0.1	254 ± 20
G10M5	77.5 ± 0.2	5.7 ± 0.5	443 ± 15
RPUF[a]	30.0 ± 0.2	3.8 ± 0.5	221 ± 13
EPSF[b]	40.0 ± 0.2	2.5 ± 0.1	155 ± 15

[a] RPUF refers to the rigid polyurethane foam. [b]EPSF refers to the expanded polystyrene foam.

Table S3. Thermal conductivities of the aerogels

**Sample**	**Thermal conductivity (mW m^{-1} K^{-1})**
G10	42.0 ± 0.5
G10M2.5	38.0 ± 0.8
G10M5	30.8 ± 0.7

Table S4. Density and mechanical properties of G10M5 after being soaked in water at 90 ºC and dried at 60 ºC 6 times

Times	Modulus (MPa)
0th	5.5 ± 0.5
1st	5.7 ± 0.1
2nd	7.4 ± 0.3
3rd	7.3 ± 0.2
4th	6.4 ± 0.4
5th	6.0 ± 0.5

Table S5. Density and mechanical properties of G10M5 after being soaked in water at 90 ºC and dried at 60 ºC for 6 times

Solvents	Modulus (MPa)
Water	5.9 ± 0.5
pH=1-2	5.5 ± 0.3
pH=13-14	4.0 ± 0.4
DMF	4.2 ± 0.2
ETOH	6.3 ± 0.4
PE	4.8 ± 0.3

Table S6. Characteristic TGA data of aerogels in nitrogen

**Samples**	$T_{d5\%}$ (℃)	T_{dmax} (℃)	**dW/dT (%/min)**	**Residue (%)**
MF	134.3	415	12.0	22.7
G10	267.6	336.0	6.3	27.4
G10M2.5	239.8	300.5	6.1	26.2
G10M5	235.2	318.8	8.8	26.8

Table S**7.** LOI and UL-94 test results of aerogels

Samples	LOI (%)	UL-94
G10	30.0 ± 0.5	NR
G10M2.5	31.0 ± 0.5	V-0
G10M5	36.5 ± 0.5	V-0

Table S8. Cone calorimetric data for aerogels at a heat flux of 50 kW/m^2

Samples	TTI (s)	PHRR (kW/m^2)	THR (MJ/m^2)	SPR (m^2/s)	TSP (m^2)	Residue (%)
G10	5	406.3	8.1	0.036	0.39	12.5
G10M2.5	5	314.6	8.9	0.005	0.28	12.3
G10M5	6	216.3	9.9	0.056	0.10	17.5
